# Antitumor efficacy and safety profile of gallic acid in the murine sarcoma 180 model

**DOI:** 10.1007/s12032-026-03455-8

**Published:** 2026-10-08

**Authors:** Greice Itamaro Heiden, Matheus Antoni da Silva Costa, Caroline Alfaia Silva, Felipe Mendes de Andrade de Carvalho, Ricardo Guimarães Amaral, Elena Riet Correa Rivero, Filipe Modolo, Rogério Gondak, Ricardo Luiz Cavalcanti de Albuquerque-Júnior

**Affiliations:** 1 Graduate Program in Dentistry, Federal University of Santa Catarina, Florianópolis, SC Brazil; 2 School of Biomedicine, Tiradentes University, Aracaju, SE Brazil; 3 Department of Medicine, Federal University of Sergipe, Lagarto, SE Brazil; 4 Department of Pathology, Health Sciences Center, Federal University of Santa Catarina, Florianópolis, SC Brazil; 5 Health Sciences Center, Federal University of Santa Catarina, Florianópolis, Santa Catarina 88040-900 Brazil

**Keywords:** Gallic acid, 5-fluorouracil, Antitumor, Sarcoma 180 model, Apoptosis

## Abstract

Gallic acid (GA) is a plant-derived polyphenol with recognized anticancer activity, but in vivo evidence, especially in mesenchymal solid tumors, remains limited. This study investigated the antitumor effects and mechanistic pathways of GA in the murine Sarcoma 180 (S-180) model. Fifty male Swiss mice bearing S-180 tumors received oral GA (10, 25, or 50 mg/kg/day), 5-fluorouracil (5-FU; 25 mg/kg, intraperitoneal), or vehicle for seven days. Tumor weight and growth inhibition were assessed, along with histopathology and immunohistochemistry for proliferation (Ki-67, a proliferation marker), apoptosis (cleaved caspase-3 and caspase-8, Bcl-2-associated X protein [Bax], and B-cell lymphoma 2 [Bcl-2]), nuclear factor kappa B (NF-κB), and systemic toxicity markers (hematological, hepatic, and renal). GA at 50 mg/kg significantly reduced tumor weight and proliferation, decreased Ki-67 and NF-κB expression, increased Bax, reduced Bcl-2, and enhanced cleaved caspase-3, indicating predominant activation of the intrinsic (mitochondrial) apoptotic pathway. Caspase-8 levels remained unchanged. Although 5-FU produced greater tumor inhibition, it induced leukopenia and splenic atrophy, whereas GA preserved hematological, hepatic, and renal parameters. These findings support GA as a promising candidate for adjuvant cancer therapy.

**Supplementary Information:**

The online version contains supplementary material available at https://doi.org/10.1007/s12032-026-03455-8.

## Introduction

Cancer remains one of the leading causes of morbidity and mortality worldwide [1]. According to global estimates, approximately 19.3 million new cases and 10 million cancer-related deaths were reported in 2020. In Brazil alone, for each year of the 2023–2025 triennium, an estimated 704,000 new cancer cases are expected [2]. Despite advances in diagnosis and treatment, chemotherapy continues to be the primary therapeutic strategy for most cancer types [3]. However, this approach, based on the use of alkylating agents, mitotic inhibitors, or cellular metabolism inhibitors [4], is frequently associated with poor therapeutic response rates, the development of chemoresistance, and a wide range of adverse effects such as alopecia, nausea, vomiting, diarrhea, and immunosuppression [5].

Considerable attention has been directed toward the study of plant-derived natural compounds with anticancer properties, due to their lower toxicity profiles and promising therapeutic potential [6]. Among these, GA, a naturally occurring polyphenolic compound obtained from the hydrolysis of plant tannins, has emerged as a molecule of interest due to its wide-ranging biological effects, including anticancer activity [7]. In vitro studies have demonstrated the cytotoxic effects of GA against diverse tumor cell lines, such as H446 (small-cell lung carcinoma) [8], K562 (gastric adenocarcinoma) [9], A549 (non-small cell lung carcinoma) [10–12], HL60 (promyelocytic leukemia) [13], MCF-7 (breast adenocarcinoma) [14–16], and glioma cell lines U251 and U87MG [7, 17].

GA has been shown to modulate key cellular signaling pathways involved in cancer progression, including apoptosis, cell cycle arrest, angiogenesis, metastasis, migration, and oncogene expression [7]. In HeLa cells, GA (IC₅₀ = 10.00 ± 0.67 µg/mL) induces sub-G1 cell cycle arrest, inhibits cell proliferation, upregulates p53 and Bax, downregulates Bcl-2, and activates caspases-8 and − 9, indicating the simultaneous activation of intrinsic and extrinsic apoptotic pathways [18]. Similarly, activation of the p53/Bax signaling axis has been associated with the cytotoxic effects of GA, which are further enhanced when combined with doxorubicin [19]. Although these studies have substantially advanced the understanding of GA-mediated anticancer mechanisms, they have been conducted predominantly in vitro using epithelial or hematological tumor cell lines.

Consequently, important questions remain regarding whether these molecular mechanisms are reproduced in vivo, where tumor progression is influenced by the host microenvironment and systemic immune responses. While previous studies have documented the antineoplastic properties of crude plant extracts containing GA [6, 7], the specific effects of orally administered, purified GA on mesenchymal solid tumors remain largely unexplored. Furthermore, a comprehensive in vivo comparison mapping its apoptotic pathways and systemic toxicity profile against standard chemotherapy is lacking. Therefore, the present study aimed to evaluate the dose-dependent antitumor activity of purified GA in the murine Sarcoma 180 (S-180) model. By providing an integrated characterization of its mechanisms of action, specifically evaluating tumor cell proliferation, intrinsic apoptosis, and NF-κB signaling, alongside a detailed assessment of hematological, hepatic, and renal toxicity compared to 5-fluorouracil (5-FU), this study highlights key mechanistic differences from previous in vitro data and establishes novel preclinical evidence regarding the safety and efficacy of GA as an adjuvant anticancer agent.

## Materials and methods

### In vivo antitumor activity assay

The in vivo experimental procedures were previously approved by the institutional Animal Use Ethics Committee (approval #010824). Fifty adult male Swiss mice (*Mus musculus*, weighing 28 ± 5 g) were housed in polypropylene cages containing wood shavings as bedding, which was changed daily. Environmental conditions were strictly regulated, with a constant temperature of 22 ± 3 °C, relative humidity between 30% and 70%, and a 12-hour light/dark photoperiod. Filtered water and a standard commercial rodent feed (Labina^®^, Purina, São Paulo, Brazil) were provided *ad libitum*.

The transplantable S-180 tumor was originally obtained from the Central Animal Facility of Tiradentes University (UNIT, Aracaju, SE, Brazil) and maintained in our laboratory by serial in vivo passages. S-180 ascitic cells were harvested from a donor mouse previously anesthetized with 1.5% isoflurane. A 0.5 mL volume of ascitic fluid was diluted in 5 mL of Ringer’s lactate solution supplemented with gentamicin (5 mg/mL; 0.2 mL). The suspension was centrifuged at 770 × *g* for 10 min, and the resulting pellet was stained with 0.025% Trypan Blue to determine cell viability. Viable cells were counted using a Neubauer chamber and the concentration was adjusted to 2 × 10⁶ cells in 500 µL for subcutaneous injection into the axillary region of recipient mice.

Subsequently, the animals were randomly assigned into five groups (*n* = 10) according to the treatment protocols outlined in Table 1. GA (≥ 97.5% purity; Sigma-Aldrich, St. Louis, MO, USA; product code G7384, CAS 149-91-7) was dissolved in dimethyl sulfoxide (DMSO) to prepare a 100 mg/mL stock solution. Working solutions were prepared daily by diluting the stock in distilled water to final concentrations of 1.0, 2.5 and 5.0 mg/mL, corresponding to doses of 10, 25 and 50 mg/kg, respectively, given a gavage volume of 10 mL/kg. For each group (*n* = 10), the total volume required was 2.8 mL (0.28 mL per mouse), and 3.36 mL of solution was prepared to account for ~ 20% extra volume to compensate for pipetting losses. Animals were weighed immediately prior to dosing, and individual gavage volumes were calculated as 10 mL/kg × body weight (kg). Final DMSO concentrations in the gavage solutions did not exceed 5% v/v (Supplementary Table 1). Vehicles were used as controls. All solutions were freshly prepared each day and protected from light.

**Table 1:** Experimental groups according to treatment

Groups (*n* = 10)	Treatment
Vehicle	Saline, 0.28 mL/day (i.p.) and 0.28 mL of water with 5% DMSO (p.o.)
5-FU	5-fluorouracil (5-FU), 25 mg/kg/day (0.28 mL of 2.5 mg/mL solution, i.p.) and 0.28 mL of water with 5% DMSO (p.o.)
GA10	Saline, 0.28 mL/day (i.p.) and 10 mg/kg/day of GA (p.o.)
GA25	Saline, 0.28 mL/day (i.p.) and 25 mg/kg/day of GA (p.o.)
GA50	Saline, 0.28 mL/day (i.p.) and 50 mg/kg/day of GA (p.o.)

Treatments were initiated 24 h after tumor inoculation and administered once daily for seven consecutive days. On the eighth day, animals were anesthetized with a ketamine/xylazine solution (1:1, 5 mg/kg, i.p.) for blood collection (1 mL from the retro-orbital plexus) and subsequently euthanized using a higher dose of the same anesthetic mixture (20 mg/kg, i.p.). Blood samples were divided into EDTA-coated tubes for hematological testing and plain tubes for biochemical analysis. Tumor masses, liver, kidneys, and spleen were dissected, weighed (expressed in mg/100 g of body weight), and fixed in 10% buffered formalin (pH 7.4) for histological evaluation. The percentage of tumor growth inhibition was calculated according to Eq. 1.

1 $$\mathbf{T}\mathbf{G}\mathbf{i}\:\mathbf{\%}\:=\:\frac{\boldsymbol{T}\boldsymbol{W}\boldsymbol{s}\boldsymbol{a}\boldsymbol{l}-\boldsymbol{T}\boldsymbol{W}\boldsymbol{e}\boldsymbol{a}}{\boldsymbol{T}\boldsymbol{W}\boldsymbol{s}\boldsymbol{a}\boldsymbol{l}}\mathrm{I}\:\mathrm{x}\:100$$

Where: TGi (%) is the percentage of tumor growth inhibition; TWsal is the mean tumor weight (mg/100 g) of the Vehicle group; and TWea is the tumor weight (mg/100 g) of each animal of the other groups.

### Histopathological evaluation of excised tumors

All excised tumors and organs were processed using standard histological techniques and embedded in paraffin. Six histological Sections. (5 μm thick), stained with hematoxylin and eosin (H&E), were obtained from each tumor specimen for descriptive microscopic evaluation. Tumor sections were examined for growth pattern (infiltrative vs. expansive), cytological atypia (mild, moderate, or severe), and morphological features of aggressiveness, including intravascular and perineural invasion.

### Assessment of the immunohistochemical expression of cell proliferation (Ki67), apoptosis (active caspase-3, caspase 8, Bcl-2 and Bax) and NF-κB signaling

For immunohistochemistry, tumor sections  (3 μm) were mounted on silanized slides, deparaffinized with Leica DEWAX (72 °C), rehydrated through absolute ethanol, and rinsed in deionized water. Antigen retrieval was performed in sodium citrate buffer (microwave, 100 °C, 20 min), and endogenous peroxidase was blocked with 30% hydrogen peroxide (10 min, 25 °C). Sections were incubated with primary antibodies (four sections per antibody; Supplementary Table 2) at room temperature, followed by biotinylated secondary antibody and streptavidin–HRP complex (LSAB System Kit, Dako) for 1 h. Detection was achieved with 3,3′-diaminobenzidine (Sigma), counterstained with Meyer’s hematoxylin (1 min), dehydrated, cleared in xylene, and mounted with Permount (Fisher Scientific).

For quantitative analysis of immunostained cells, 10 histological fields per slide (400 ×; 0.0625 mm²) were randomly selected in a systematic pattern and photomicrographed. Immunohistochemical reactions were evaluated independently by two blinded observers under light microscopy. For nuclear markers (Ki-67 and NF-κB/p65), the percentage of positively stained nuclei was determined in five high-power fields (400×) within the most reactive areas. The labeling index was calculated as the ratio of positive nuclei to total nuclei ×100. Cytoplasmic markers (cleaved caspase-3, cleaved caspase-8, Bcl-2, and Bax) were evaluated semiquantitatively, considering both the intensity and the extent of immunostaining. Intensity was graded from 0 (absent) to 3 (strong), while extent was categorized into four groups according to the proportion of positive cells: 0 (< 5%), 1 (5–25%), 2 (26–50%), and 3 (> 50%). The final score was obtained by multiplying intensity and extent.

### Systemic toxicity assessment

Animals were monitored daily for body weight, food, and water intake as indicators of general health and potential systemic toxicity. Previously collected blood samples were analyzed for hematological (total leukocytes, lymphocytes, neutrophils, monocytes) and biochemical (AST, ALT, urea, creatinine) parameters. Hematology was performed using an automated analyzer (ABX Pentra XL 80, Biosystems, Recife, Brazil). Biochemical analyses were carried out with commercial colorimetric kits (Labtest, Lagoa Santa, Brazil), with initial readings on a semi-automated analyzer (Bioplus 200, Barueri, Brazil) and confirmation using a fully automated system (Labmax 240, Centerlab, Belo Horizonte, Brazil). Additionally, the visceral organs (kidneys, liver, and spleen) were collected after euthanasia, weighed, and fixed in 10% neutral buffered formaldehyde (pH 7.4). The samples were then routinely processed and embedded in paraffin for further histological examination.

### Statistical analysis

Normality was assessed using the Shapiro–Wilk test and homoscedasticity with Bartlett’s (parametric data) or Levene’s test (nonparametric data). Parametric data were expressed as mean ± SD and analyzed by one-way ANOVA with Tukey’s post hoc test. Nonparametric data were reported as median (IQR, min–max) and analyzed by Kruskal–Wallis followed by Dunn’s post hoc test. Statistical significance was set at *p* < 0.05. Analyses were performed using GraphPad Prism 5.0 (San Diego, CA, USA).

## Results

**Fig. 1:**
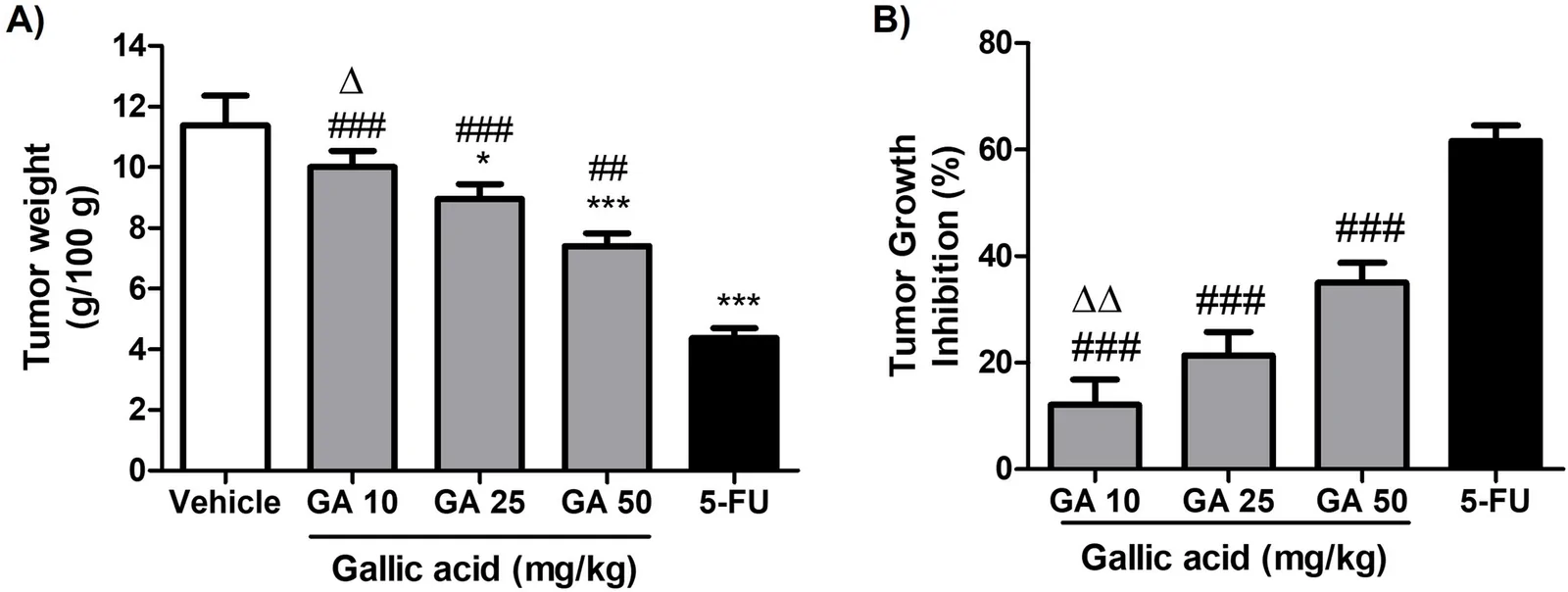
**A** Tumor weight (g/100 g) in groups: Vehicle, GA10/25/50 (10, 25, 50 mg/kg gallic acid), and 5-FU (25 mg/kg). **B** Tumor growth inhibition (%) relative to Vehicle group. Significance: vs. Vehicle (*p < 0.05, ***p < 0.001); vs. 5-FU (##*p* < 0.01, ####*p* < 0.001); vs. GA50 (∆*p* < 0.05, ∆∆*p* < 0.01); one-way ANOVA with Tukey’s post hoc test

Treatment with GA at 25 and 50 mg/kg significantly reduced tumor weight to 9.38 ± 0.49 and 7.21 ± 0.42 g/100 g, respectively (*p* < 0.05 and *p* < 0.001) compared with the Vehicle group (11.49 ± 0.97 g/100 g). The 5-FU group showed the greatest tumor reduction (4.28 ± 0.33 g/100 g), which was significantly lower than that observed in GA25 and GA50. Tumor growth inhibition percentages were 17.57 ± 4.37% and 33.67 ± 3.75% for GA25 and GA50, respectively, and 62.98 ± 2.33% for 5-FU. No significant antitumor effect was observed with GA at 10 mg/kg, which showed a tumor weight of 9.65 ± 0.53 g/100 g and growth inhibition of 18.18 ± 4.71% (Fig. 1). Based on the antitumor activity results, the 50 mg/kg dose, which exhibited the greatest antitumor efficacy, was selected for all subsequent hematological, biochemical, histopathological, and mechanistic analyses as a methodological optimization strategy, allowing a more comprehensive characterization of its biological effects while reducing the number of experimental animals and laboratory analyses.

**Fig. 2:**
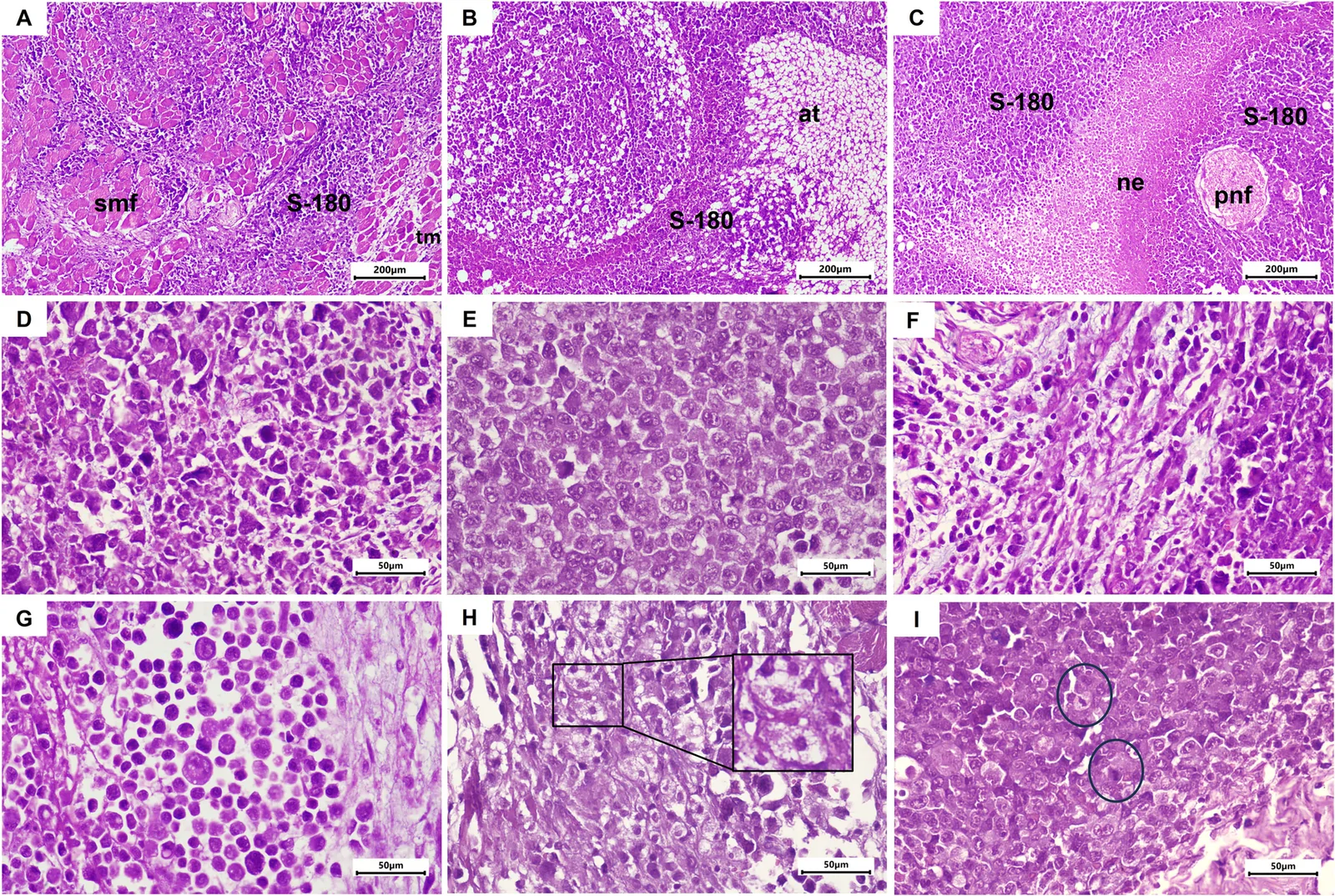
Histological slides represent the main histological features of S-180. **A** muscular, (**B**) adipose and (**C**) perineural invasion by tumor cells (H&E, 100 x). Tumor cells displaying (**D**) rhabdoid, (**E**) epithelioid, (**F**) spindle-shaped, (**G**) leukemoid and (**H**) “plant-like” appearance (H&E, 400 x). **I** Mitosis figures (H&E, 400 x). Smf – skeletal striated muscle; at – adipose tissue; pnf – peripheral nerve fibers; S-180 – S-180 tumor cells; ne – necrosis

Histologically, S-180 tumors exhibited similar baseline features across groups, including solid and diffuse neoplastic proliferation, infiltration of skeletal muscle and adipose tissue, perineural invasion, and extensive coagulative necrosis. Necrotic areas were smaller in the 5-FU and GA50 groups. Tumor cells exhibited marked morphological heterogeneity. The majority were polygonal with abundant eosinophilic cytoplasm and central or eccentric dark nuclei (rhabdoid appearance). Epithelioid cells with large nuclei, dispersed chromatin, and occasional prominent nucleoli were frequent. Less common were spindle-shaped cells in fascicular arrangements, and small rounded cells with scant cytoplasm and centric nuclei resembling a leukemoid pattern. A subset of large cells with vacuolated, granular cytoplasm showing irregular eosinophilic septa (“plant-like cells”) was also observed. Mitotic activity was consistently high (> 4 mitoses/10 fields, 400×) (Fig. 2).

**Fig. 3:**
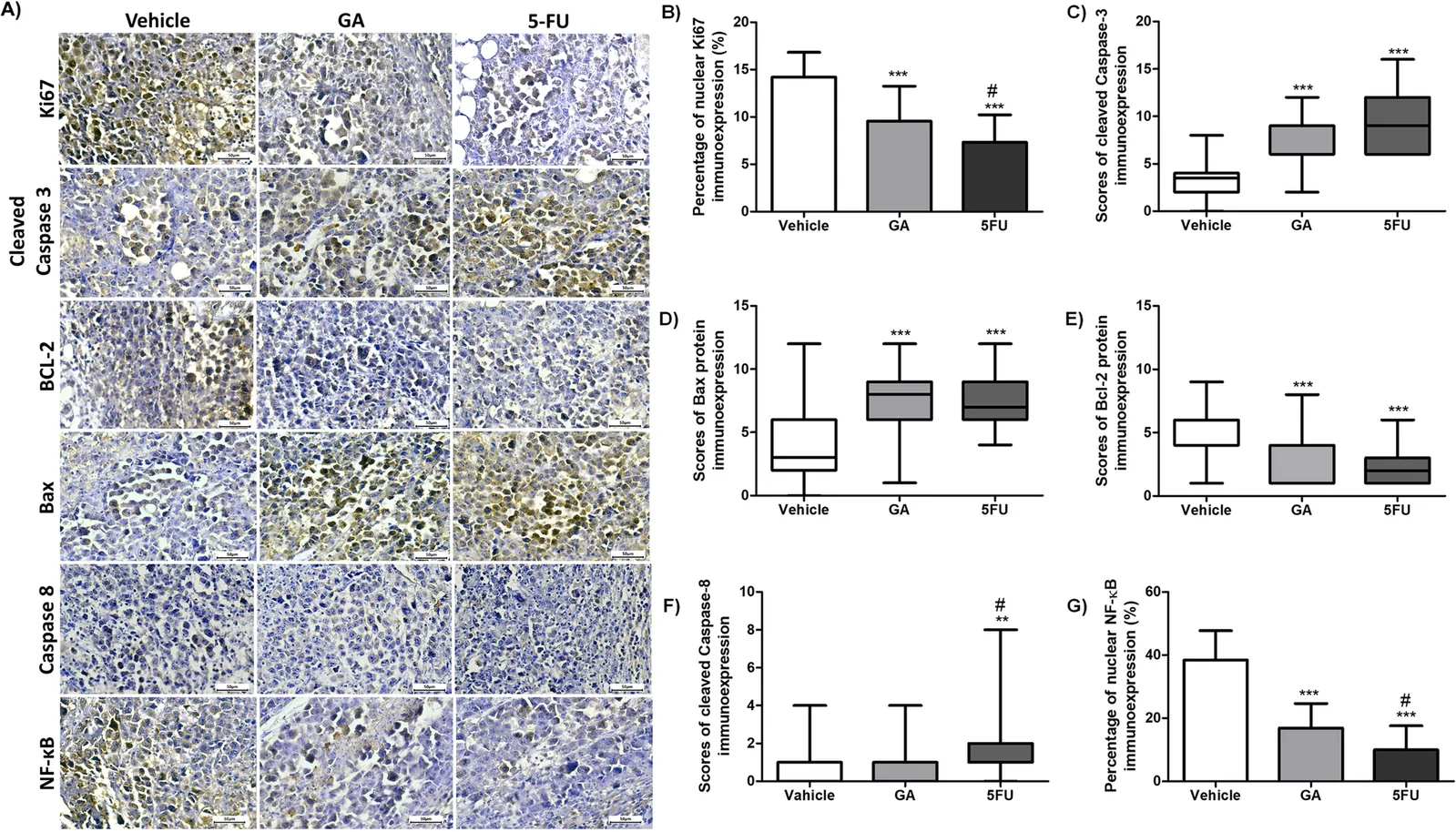
Immunohistochemical expression profile of all the antigens analyzed in tumor tissues across experimental groups. **A** Representative photomicrographs showing nuclear expression pattern for Ki-67 and NF-κB, and cytoplasmic expression for cleaved caspase-3, Bcl-2, Bax, and cleaved caspase-8 (SABC, 400×). Quantitative and semi-quantitative analysis for (**B**) Ki-67, (**C**) cleaved caspase-3, (**D**) Bax, (**E**) Bcl-2, (**F**) cleaved caspase-8, and (**G**) NF-κB. Significance: vs. Vehicle - **p < 0.01 and ***p < 0.001; vs. GA (50 mg/kg) - #*p* < 0.05 (B and G: data expressed as mean ± SD; ANOVA and Tukey’s multiple comparisons test; C, D, E, and F: data expressed as median with IQR, Kruskal-Wallis test and Dunn’s multiple comparisons test)

As shown in Fig. 3, Ki67 and NF-κB displayed nuclear immunoexpression, whereas cleaved caspase-3, cleaved caspase-8, Bcl-2, and Bax exhibited cytoplasmic staining. Treatment with GA significantly reduced Ki67 levels (9.5 ± 3.6%; *p* < 0.001) and increased cleaved caspase-3 expression (9.0, 2.0–4.0; *p* < 0.001) compared with the Vehicle group (14.2 ± 2.6% and 3.0, 2.0–4.0, respectively), indicating an overall shift toward tumor cell death (proliferation vs. apoptosis). GA also significantly increased Bax expression (8.0, 6.0–9.0; *p* < 0.001) while reducing Bcl-2 (1.0, 1.0–4.0; *p* < 0.001) and NF-κB (14.4 ± 7.9%; *p* < 0.001) compared with Vehicle (Bax = 3.0, 2.0–6.0; Bcl-2 = 4.0, 4.0–6.0; NF-κB = 38.4 ± 9.3%). However, GA and Vehicle did not differ significantly in cleaved caspase-8 immunoexpression (*p* > 0.05).

All the immunohistochemical markers evaluated in the 5-FU group differed significantly from the Vehicle group (*p* < 0.05). Furthermore, in comparison with the GA group, the 5-FU group showed significantly lower immunoexpression of the antigens Ki67 (7.3 ± 2.8%; *p* < 0.05) and NF-κB (9.9 ± 7.6%; *p* < 0.05), and greater expression of caspase-8 (1.0; 1.0–2.0; *p* < 0.05).

Erythrocyte indices did not differ significantly among groups (*p* > 0.05). The 5-FU group showed a marked reduction in total leukocyte counts (2.07 ± 0.83 × 10³/mm³; *p* < 0.01), consistent with myelosuppression, while platelet counts remained within normal limits across treatments (*p* > 0.05). ALT levels were similar among groups (*p* > 0.05), whereas AST was significantly lower in the 5-FU group compared with Vehicle (97.66 ± 28.33 U/L; *p* < 0.01). Urea and creatinine values stayed within reference ranges, indicating preserved renal function (Table 2).

**Table 2:** Hematological and Biochemical Parameters of Swiss Mice with S-180 Subjected to Treatment with Vehicle, GA (50 mg/kg) and 5-FU (25 mg/kg)

Parameter	Vehicle	GA	5-FU	Reference Range¹
Erythrocytes (10⁶/mm³)	7.74 ± 1.32	7.88 ± 1.75	7.74 ± 1.32	7.2–10.3
Hematocrit (%)	43.94 ± 9.22	45.43 ± 12.50	43.62 ± 11.86	35.4–51.2
MCV (fL)²	56.30 ± 1.73	55.98 ± 1.62	52.53 ± 3.63	49–58
Total Hemoglobin (g/dL)	14.40 ± 2.92	15.06 ± 3.59	13.88 ± 3.29	11.8–16.5
MCH (pg)³	17.30 ± 1.23	18.67 ± 0.60	17.60 ± 2.31	13.6–18.6
MCHC (g/dL)⁴	33.92 ± 2.48	33.30 ± 1.79	35.08 ± 5.97	30.4–36.4
Total Leukocytes (×10³/mm³)	4.91 ± 1.66	4.73 ± 1.29	**2.07 ± 0.83**⁎⁎	3.4–9.8
Platelets (×10³/mm³)	871.03 ± 203.22	735.52 ± 222.23	693.67 ± 179.83	491.0–1170.0
Alanine Aminotransferase (ALT, U/L)	133.33 ± 22.77	92.98 ± 23.52	93.51 ± 15.93	70.0–140.0
Aspartate Aminotransferase (AST, U/L)	141.15 ± 23.79	106.33 ± 18.43	**97.66 ± 28.33**⁎⁎	73.3–188.0
Urea (mg/dL)	46.69 ± 9.37	46.56 ± 9.31	43.46 ± 9.55	20.0–65.0
Creatinine (mg/dL)	0.34 ± 0.14	0.30 ± 0.05	0.31 ± 0.05	0.2–0.4

(1) Araújo et al., [20]

(2) MCV – mean corpuscular volume

(3) HCM – mean corpuscular hemoglobin

(4) MCHC – mean corpuscular hemoglobin concentration

Significant differences compared to the Vehicle group are expressed as **p < 0.01 (ANOVA and Tukey’s multiple comparison test)

**Fig. 4:**
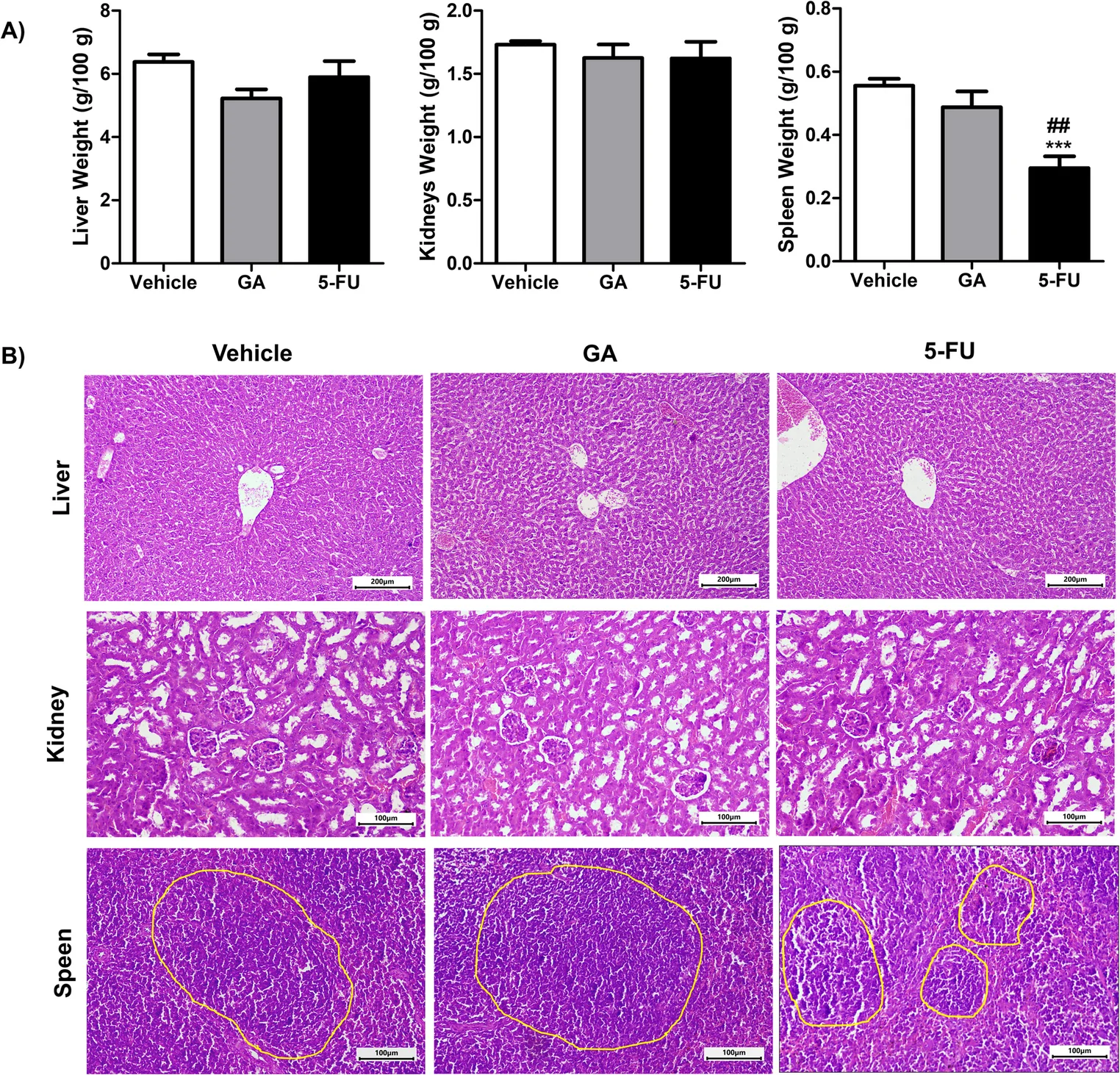
**A** Mean weight (g/100 g) of the liver, kidneys and spleen of experimental animals. Significance: Vs. Vehicle - ***p < 0.001; Vs. GA - ##*p* < 0.01 (ANOVA and Tukey post-hoc multiple comparison test). **B** Photomicrographs of histological sections (H&E, 100 ×) representing the main morphological findings of the organs (liver, kidneys, and spleen) of experimental animals from different groups (Vehicle, GA at 50 mg/kg, and 5-FU). Liver and kidneys show typically normal histology in all the groups, whereas only the 5-FU group showed architectural disorganization and follicular atrophy (yellow) of the spleen

No significant differences were found in liver or kidney weights among the experimental groups (*p* > 0.05). In contrast, the 5-FU group exhibited a significant reduction in spleen weight compared with the GA (*p* < 0.05) and Vehicle (*p* < 0.001) groups (Fig. 4A). Histological evaluation revealed normal liver and kidney architecture, whereas spleens from the 5-FU group showed white pulp atrophy with reduced follicular diameter and disrupted lymphoid organization (Fig. 4B).

## Discussion

GA exhibits multiple biological effects linked to anticancer activity [7]. However, despite significant in vitro advances, in vivo evidence remains limited, particularly regarding the molecular mechanisms underlying GA’s antitumor action. Over recent decades, murine models have been used to assess therapeutic efficacy, identify biomarkers, and explore tumor biology [21]. Among these, the S-180 model is widely used because of its efficient implantation procedure and rapid tumor growth, which allow for swift experimental execution [22].

Tumor weight and growth inhibition rates are key indicators of antitumor activity in vivo [23–26]. In the present study, analysis of tumor weight and growth inhibition demonstrated that GA exhibits significant, dose-dependent antitumor activity in the murine S-180 model. Although this is the first study to evaluate GA using the S-180 model, the findings align with previous in vivo research confirming its antineoplastic effects in various xenograft tumor models [27, 28].

Although the mechanisms underlying the cytotoxic action of GA against tumor cells are not yet fully elucidated, evidence suggests that GA modulates several biological signaling pathways, including those involved in cell cycle regulation and apoptosis [7].

In this study, GA significantly reduced Ki-67 immunoexpression, indicating a decrease in the proliferative activity of S-180 cells [29, 30]. Quantitative assessment of Ki-67 is a well-established approach for evaluating tumor growth and has been successfully applied in S-180 models [24, 25]. These findings suggest that the antitumor activity of GA is associated, at least in part, with suppression of tumor cell proliferation, consistent with previous reports demonstrating that GA blocks cell-cycle progression through inhibition of the PI3K/Akt pathway, downregulation of cyclin D1, upregulation of p21 and p27, and consequent G1/S cell-cycle arrest [31, 32]. Nevertheless, reduced Ki-67 immunostaining should be interpreted cautiously, as it may reflect both inhibition of cell proliferation and increased elimination of proliferating cells through apoptosis [33].

To further investigate this possibility, apoptotic markers were evaluated. GA significantly increased cleaved caspase-3 immunoexpression, consistent with enhanced apoptotic activity in vivo and corroborating previous in vitro evidence of its pro-apoptotic effects [18, 34–37]. Moreover, the concomitant increase in Bax and reduction in Bcl-2, together with the absence of significant changes in cleaved caspase-8, are consistent with preferential activation of the intrinsic (mitochondrial) apoptotic pathway. This immunohistochemical profile is compatible with a shift in the balance of the Bcl-2 family toward a pro-apoptotic state, which may favor mitochondrial membrane permeabilization and subsequent activation of the caspase cascade. In contrast, the unchanged expression of cleaved caspase-8 suggests that the extrinsic apoptotic pathway is unlikely to be the predominant mechanism involved in the antitumor effects of GA in this model [38, 39].

Collectively, these findings indicate that the antitumor activity of GA in the S-180 model involves complementary mechanisms, including suppression of tumor cell proliferation and induction of mitochondrial-mediated apoptosis. The integration of these effects provides mechanistic support for the pronounced tumor growth inhibition observed in vivo and reinforces the therapeutic potential of GA as an anticancer agent.

GA also significantly reduced NF-κB immunoexpression, suggesting suppression of a key signaling pathway involved in tumor cell survival and proliferation. Given the central role of NF-κB in regulating anti-apoptotic proteins and cell-cycle progression, its inhibition is consistent with the increased apoptosis and reduced proliferative activity observed in the present study [40]. This finding agrees with previous reports demonstrating that GA inhibits the MAPK/NF-κB signaling pathway [41], possibly through reduced phosphorylation and altered acetylation of the p65 subunit, leading to decreased NF-κB transcriptional activity [42]. Thus, inhibition of NF-κB likely represents an additional mechanism by which GA shifts the balance toward apoptosis in the S-180 model.

Overall, the present findings indicate that the antitumor activity of GA is associated with complementary mechanisms involving suppression of tumor cell proliferation, inhibition of NF-κB signaling, and preferential activation of the intrinsic apoptotic pathway. Although GA significantly reduced tumor growth, its antitumor efficacy remained lower than that of 5-FU. This difference may be explained by the more pronounced inhibition of Ki-67 and NF-κB observed with 5-FU, together with evidence of caspase-8 activation, suggesting engagement of both the intrinsic and extrinsic apoptotic pathways. These mechanistic differences may account, at least in part, for the greater antitumor activity of 5-FU in the S-180 model.

Hematological analyses showed that GA preserved leukocyte counts, while 5-FU induced leukopenia and splenic white pulp atrophy, reflecting myelosuppression [23–26], and minor fluctuations in erythrocyte parameters were considered physiological [43]. Hepatic and renal functions remained intact, with serum AST, ALT, urea, and creatinine within normal ranges and no histopathological alterations observed [44]; the decrease in AST with GA at 50 mg/kg may reflect reduced tumor growth and associated muscle damage [28]. Collectively, these findings suggest that GA is a promising adjuvant in chemotherapy, capable of mitigating hematological adverse effects while preserving hepatic and renal function.

Taken together, these findings demonstrate that GA exerts significant antitumor activity in the murine S-180 model, which is associated with reduced tumor cell proliferation, preferential activation of the intrinsic apoptotic pathway, and decreased NF-κB expression, suggesting inhibition of this pro-survival signaling pathway. In addition, GA preserved hematological, hepatic, and renal parameters, in contrast to 5-FU, which induced immunosuppressive effects. Collectively, these results indicate that GA combines significant antitumor activity with a favorable safety profile, supporting its potential as a promising candidate for adjunctive cancer therapy.

## Supplementary Information

Supplementary Material 1.

Supplementary Material 2.

## Data Availability

Study data are available from the corresponding authors on reasonable request.
