# Supplementary material for: Antitumor efficacy and safety profile of gallic acid in the murine sarcoma 180 model
Source: Med Oncol. 2026 Oct 8;43(11):331. doi: 10.1007/s12032-026-03455-8 (PMC13649940; doi:10.1007/s12032-026-03455-8)
Supplement: Supplementary file 1 [file 12032_2026_3455_MOESM1_ESM.docx]

**Supplementary Table 1.** Preparation of gallic acid working solutions for oral gavage in Swiss mice (10, 25, and 50 mg/kg doses)

| Dose (mg/kg) | Final conc. (mg/mL) | Total required (for 3.36 mL)* | Stock (100 mg/mL) | Water to add (mL) | Final % DMSO |
| --- | --- | --- | --- | --- | --- |
| 10 | 1.0 | 3.36 mg | 33.6 µL | 3.3264 mL | ~1.0% |
| 25 | 2.5 | 8.40 mg | 84.0 µL | 3.2760 mL | ~2.5% |
| 50 | 5.0 | 16.8 mg | 168 µL | 3.1920 mL | ~5.0% |

* Volume prepared per group = 0.28 mL × 10 mice = 2.8 mL - with 20% overage = 3.36 mL
