# Supplementary material for: Antitumor efficacy and safety profile of gallic acid in the murine sarcoma 180 model
Source: Med Oncol. 2026 Oct 8;43(11):331. doi: 10.1007/s12032-026-03455-8 (PMC13649940; doi:10.1007/s12032-026-03455-8)
Supplement: Supplementary file 2 [file 12032_2026_3455_MOESM2_ESM.docx]

**Supplementary Table 2.** Primary antibodies used for immunohistochemical analysis.

| Target Antigen | Supplier | Catalog Number | Specificity / Clone | Dilution |
| --- | --- | --- | --- | --- |
| Ki-67 | ELK Biotechnology | EM1064 | Mouse monoclonal | 1:200 |
| Cleaved Caspase-3 | ELK Biotechnology | EM1188 | Mouse monoclonal | 1:200 |
| Cleaved Caspase-8 | ELK Biotechnology | ES8370 | Rabbit polyclonal | 1:50 |
| Bax | ELK Biotechnology | EM1268 | Mouse monoclonal (6F11) | 1:200 |
| Bcl-2 | ELK Biotechnology | EM1041 | Mouse monoclonal | 1:200 |
| NF-κB (p65) | ELK Biotechnology | EM1111 | Mouse monoclonal | 1:200 |

**Abbreviations:** Bax, Bcl-2-associated X protein; Bcl-2, B-cell lymphoma 2; NF-κB, nuclear factor kappa B.
